# Effects of the lysosomal destabilizing drug siramesine on glioblastoma in vitro and in vivo

**DOI:** 10.1186/s12885-017-3162-3

**Published:** 2017-03-07

**Authors:** Stine S. Jensen, Stine A. Petterson, Bo Halle, Charlotte Aaberg-Jessen, Bjarne W. Kristensen

**Affiliations:** 1 Department of Pathology, Odense University Hospital, Winsløwparken 15, 3. floor, 5000 Odense C, Denmark; 2 Institute of Clinical Research, University of Southern Denmark, Winsløwparken 19.3, 5000 Odense C, Denmark; 3 Department of Neurosurgery, Odense University Hospital, Sdr. Boulevard 29, 5000 Odense C, Denmark

**Keywords:** Siramesine, Glioblastoma, Cancer stem cell, Lysosomes, Spheroids, Brain slice cultures

## Abstract

**Background:**

Glioblastoma is the most frequent and most malignant brain tumor with the patients having a median survival of only 14.6 months. Although glioblastoma patients are treated with surgery, radiation and chemotherapy recurrence is inevitable. A stem-like population of radio- and chemoresistant brain tumor-initiating cells combined with the invasive properties of the tumors is believed to be critical for treatment resistance. In the present study, the aim was to investigate the effect of a novel therapeutic strategy using the lysosomotropic detergent siramesine on glioblastomas.

**Methods:**

Standard glioma cell lines and patient-derived spheroids cultures with tumor-initiating stem-like cells were used to investigate effects of siramesine on proliferation and cell death. Responsible mechanisms were investigated by inhibitors of caspases and cathepsins. Effects of siramesine on migrating tumor cells were investigated by a flat surface migration assay and by implanting spheroids into organotypic rat brain slice cultures followed by confocal time-lapse imaging. Finally the effect of siramesine was investigated in an orthotopic mouse glioblastoma model. Results obtained in vitro and in vivo were confirmed by immunohistochemical staining of histological sections of spheroids, spheroids in brain slice cultures and tumors in mice brains.

**Results:**

The results showed that siramesine killed standard glioma cell lines in vitro, and loss of acridine orange staining suggested a compromised lysosomal membrane. Co-treatment of the cell lines with inhibitors of caspases and cathepsins suggested differential involvement in cell death. Siramesine caused tumor cell death and reduced secondary spheroid formation of patient-derived spheroid cultures. In the flat surface migration model siramesine caused tumor cell death and inhibited tumor cell migration. This could not be reproduced in the organotypic three dimensional spheroid-brain slice culture model or in the mice xenograft model.

**Conclusions:**

In conclusion the in vitro results obtained with tumor cells and spheroids suggest a potential of lysosomal destabilizing drugs in killing glioblastoma cells, but siramesine was without effect in the organotypic spheroid-brain slice culture model and the in vivo xenograft model.

**Electronic supplementary material:**

The online version of this article (doi:10.1186/s12885-017-3162-3) contains supplementary material, which is available to authorized users.

## Background

The standard treatment of glioblastomas includes surgical resection, fractionated radiation and concomitant as well as adjuvant chemotherapy with temozolomide. This treatment has improved survival but despite these improvements the median survival is only 14.6 months [1]. Two biological aspects believed to be highly responsible for tumor recurrence are the resistant tumor stem-like cells [2] and the invasive properties of glioblastomas [3]. Treatments targeting the tumor stem cells and the invasive cells are therefore of great interest.

The lysosomal cell death pathway involves lysosomal membrane permeabilization, thus being a cell death pathway still functional in the tumor cells. By lysosomal membrane permeabilization the lysosomal content translocates to the cytosol and may cause programmed cell death [4, 5]. Among the proteases responsible for this cell death are the cathepsins, which are still active at neutral pH [4]. Most excitingly the cathepsins are capable of inducing a caspase-independent and mitochondrial-independent cell death promoting cell death in tumor cells with multiple defects in the classical apoptosis pathway [6].

A compound shown to accumulate in the lysosomes causing lysosomal membrane permeabilization and release of the cathepsins to the cytosol is the σ 2 receptor agonist, siramesine (Lu-28-179; 1 V-[4-[1-(4-fluorphenyl)-1H-indol-3-yl] butan-1-yl]spiro[isobenzofuran-1(3H),4 V-piperidine]). Siramesine was originally designed to treat anxiety and depression and it was shown to successfully enter the brain in mice ex-vivo binding studies [7]. The drug was well tolerated and non-toxic in humans but the effect was not satisfactory [8]. Because of the lack of side effects and the suggested role of σ 2 receptors triggering cell death, siramesine was investigated as an anti-cancer drug [9]. Indeed, siramesine induced cell death in immortalized and tumorigenic cells [9] by lysosomal leakage of cathepsins and oxidative stress [10]. Siramesine was found to directly destabilize the lysosomal membrane followed by lysosomal dysfunction leading to permeabilization of the membrane and release of cathepsins to the cytosol resulting in cathepsin mediated cell death [9, 10]. Importantly, the cell death was independent of caspases and P53 tumor suppressor protein and insensitive to the anti-apoptotic effect of Bcl-2 [9–11].

The aim of the current study was to investigate the effect of siramesine on glioblastoma cells using approaches comprising both immature tumor stem-like cells, differentiated tumor cells and migrating tumor cells. Accordingly, we used both standard glioma cell lines and patient-derived spheroids cultures with tumor-initiating stem-like cells [12, 13]. To perform a thorough testing, spheroid cultures were implanted in three dimensionel organotypic brain slice cultures [13, 14] and used for generation of patient-like tumors in a glioblastoma xenograft mice model [12, 15, 16]. Using these approaches the in vitro results with tumor cells and spheroids suggested a potential of lysosomal destabilizing drugs in killing glioblastoma cells, but in the organotypic spheroid-brain slice culture model and the in vivo xenograft model siramesine was without effect.

## Methods

### Cells and treatments

In the present study we used the commercial human glioma cell lines, U87, A172, T98G (all from European Collection of Cell Cultures (ECACC), catalogue numbers: 89081402, 88062428 and 92090213, respectively) and U251 (from CLS, cell lines service, Germany, catalogue number: 300385) cultured in serum containing medium as described earlier [12].

The glioblastoma stem cell-like containing spheroid (GSS) cultures T78, T86 and T87 were established in our laboratory and cultured under stem cell promoting conditions as neurospheres (spheroids). The spheroids were cultured in serum-free medium as described earlier [12]. The GSS cultures have the ability to form new spheroids at clonal density, a karyotype typical of glioblastomas, the ability to differentiate into cells expressing neuronal, astrocytic and oligodendrocyte markers upon culturering in serum-containing medium and the ability to form highly invasive tumors upon orthotopic xenografting [15].

Siramesine was kindly provided by H. Lundbeck A/S, Valby, Denmark. Dimethylsulfoxid (DMSO) was used as a solvent for siramesine in all in vitro studies. The cathepsin and caspase inhibitors used were z-FA-FMK (cathepsin B, L and S), Ca-074-Me (irreversible capthepsin B inhibitor), z-DEVD-FMK (caspase 3 and 7), and z-DEVD-CMK (caspase 3), all from Bachem. The inhibitors were added 1 h before the addition of siramesine. The glioma cell lines were incubated with inhibitors and siramesine for 48 h before measuring cell proliferation and viability.

### Cell proliferation and viability in adherent glioma cell lines

The cell proliferation was analysed using the cell proliferation reagent WST-1 (2-(4-Iodophenyl)-3-(4-nitrophenyl)-5-(2,4-disulfophenyl)-2H-tetrazolium, monosodium salt, from Roche) according to the manufactures instructions. After 24 or 48 h of incubation of cells with siramesine, the WST-1 reagent was added and the absorbance at 450 nm was found using an absorbance microplate reader (BioTek ELx808, Holm and Halby, Denmark).

A lactate dehydrogenase kit (LDH Cytotoxicity Detection Kit, Roche) was used to detect cell death according to the manufactures instructions, detecting LDH released from cells with a permeabilized membrane. Medium from each well in a 96-well plate was transferred to a new plate where after the LDH reaction mix were added and the absorbance measured at 450 nm in the absorbance microplate reader.

### Change in lysosomal acidity

The glioma cell lines were incubated in 12 well glass bottom plates until approximately 80% confluence and exposed to siramesine (5–30 μM). After 1 h, Acridine orange (5 μg/ml, Invitrogen) was added to the cells and incubated for 15 min at 37 °C. Images were recorded using confocal microscopy (Nikon, Inverted Microscope, ECLIPSE TE2000-E). Analysis was made using the image analysis tool Visiomorph™ (Visiopharm, Hørsholm, Denmark). The results represent the proportion of red/green + yellow area, accounting for the loss of red staining and gain of green and yellow staining in the cells.

### Cell death in GSS spheroid cultures

Cell death in the GSS spheroid cultures was analyzed using propidium iodide (PI) (2 μM, Invitrogen) and Hoechst 33342 (10 mM, Invitrogen). The spheroids were treated with siramesine for 24 h before addition of PI for 1 h followed by Hoechst for 15 min. The spheroids were then analyzed using a confocal microscope recording z-stacks which afterwards were superimposed into one image. The images were analyzed using the image analysis tool Visiomorph establishing a classifier identifying the PI uptake as red staining area and the total nuclear area as the sum of red staining and blue Hoechst staining. The area ratio between red staining and red plus blue staining was calculated.

### Secondary spheroids formation after siramesine treatment

Spheroids treated with siramesine for 24 h were dissociated, the cells were counted and 1000 cell/ml were added to each well in a six-well plate. The cells were allowed to form secondary spheroids and after 2–3 weeks the number of spheroids in each well was counted.

### Immunohistochemical staining of GSS cultures exposed to siramesine

After having analyzed the spheroids exposed to siramesine with PI uptake, the spheroids were fixed in 4% formalin for 24 h followed by paraffin embedding. Three-micrometer sections of the paraffin-embedded spheroids were cut on a microtome. Thereafter one section was hematoxylin eosin stained and adjacent sections immunohistochemically stained on a Dako autostainer, Universal Staining System. For immunohistochemical staining, paraffin sections were deparaffinized and heat-induced epitope retrieval was performed by incubation in a TEG buffer solution of 10 mmol/L Trisbase and 0.5 mmol/L EGTA (CD133, nestin, Bmi-1, Sox 2, Ki67, Lamp-2, cathepsin B) or EDTA buffer (Cathepsin B). After blocking of endogenous peroxidase activity by incubation in 1.5% hydrogen peroxide (H2O2), the sections incubated for 60 min with primary antibodies against CD133 (1 + 40, CD133/1 W6B3C1, Miltenyi Biotec), Nestin (1 + 3000, 196908, R&D Systems), Bmi-1 (1 + 400, F6, Upstate Biotechnology), Sox 2 (1 + 400, 245610, R&D Systems), Ki67 (1 + 200, MIB-1, Dako), Lamp-2 (1 + 2000, H4B4, Developmental Studies Hybridoma Bank), Cathepsin B (1 + 200, polyclonal, Abgent) and Cathepsin D (1 + 750, EPR3057Y, Epitomics). Detection of immunohistochemical staining CD133 was detected by CSAII (Catalysed Signal Amplification II kit, Dako), nestin, Bmi-1 and sox 2 with Power Vision (Dako) and Ki-67, Lamp-2, cathepsin B and cathepsin D was performed by use of the detection system EnVision (Dako). The visualization was performed using diaminobenzidine as chromogen. Finally, the sections were counterstained with Haematoxylin and Eosin (H&E) and cover slips were mounted with Aquatex. Paraffin sections of tissue microarrays with 28 normal tissues and 12 cancers were used as positive control. Primary antibody omission was used as negative control.

### Spheroid migration assay

Briefly, a reduced growth factor basement membrane matrix (Geltrex™, Life Technologies™, Denmark) solution was mixed with neurobasal medium, and added to each well in a 12-well plate. Subsequently, the supernatant was aspirated and the spheroids were placed individually (1 spheroid/well). Afterwards neurobasal medium was added to each well and cells were allowed to settle and migrate for 24 h, while being exposed to siramesine in increasing concentrations. The cells were monitored by light microscopy and imaging. The outer diameter of the migrating cells was measured using ImageJ software, relatively to spheroid diameter measured at day 0. Cell death in spheroids was visualized using PI (2 μM).

### Preparation of organotypic brain slice cultures

Newborn Wistar rat pups (Taconic Europe, Denmark) used in the present study were treated according to the procedures at the Biomedical Laboratory, University of Southern Denmark.

Organotypic corticostriatal slice cultures were prepared as previous described [13, 14]. Seven days after the start of culturing the brain slice cultures were exposed to siramesine and cell death was examined by PI uptake as described earlier by Nørregaard et al. [14].

### Preparation of co-cultures

GSS culture spheroids were implanted in the area between cortex and striatum close to corpus callosum. The medium was changed to serum-free medium before implanting the spheroids. The spheroids (200–300 μm) were incubated in DiO solution (1 mM, Molecular Probes, Invitrogen) for 24 h, before implanting them in the brain slice cultures. PI (2 μM, Molecular Probes, Invitrogen) was added to the medium to monitor cell death in spheroids and brain slices. Before start of exposure (Day 0) confocal z-stacks with 20 μm steps were recorded after 1 h of incubation. Thereafter the z-stacks were superimposed into one image representing the entire spheroid and surrounding brain tissue. This procedure was repeated at day 3 and day 6, whereafter the co-cultures were fixed in 4% formalin and paraffin embedded.

As a control assay to ensure cell death in the spheroids, DiO stained spheroids derived from T78 and T86 were placed on the same type of membranes used for the co-cultures. Cell death was investigated in the spheroids exposed to 20 μM siramesine with PI uptake recording confocal z-stacks as for the brain slice cultures. Cell death in the tumor cells was quantified using Visiomorph software. A classifier identifying PI uptake as red staining, co-expression of PI and DiO as yellow staining and DiO as green staining was created. The data were illustrated as area of cell death (red + yellow staining) divided by total spheroid area (red + yellow + green staining).

### Immunohistochemical studies of co-cultures

The fixed and paraffin embedded co-cultures were cut in three μm sections and immunhistochemical stained. Immunohistochemistry was performed as described earlier, using the antibodies CD56 (1 + 100, CO4-NCAM, Neomarkers) and Vimentin (1 + 200, EP20, Epitomics) to identify human tumor cells in the rat tissue. A panel of the stem cell markers CD133, Nestin and Podoplanin (1 + 100, D2-40, Dako) as well as the proliferation marker Ki-67 was furthermore used as described above. The staining of the stem cell markers were assessed in the implanted spheroids by semi-quantitative scoring (0, 1+, 2+ and 3+). A Ki-67 labeling index was measured using the software program Tissuemorph (Visiopharm, Hørsholm, Denmark).

### Glioblastoma tumor xenografts

The experimental procedure was performed as previously described [12]. Female Balb/c nude (BALBNU-F, Taconic) mice were anesthetized subcutaneously and placed in a stereotactic instrument. Through a burr hole a 2-μL suspension of 300,000 single cells was injected into the striatum. Mice (*n* = 42) were implanted with the standard cell line U87 (*n* = 22), and the patient-derived cell line T78 (*n* = 20).

Siramesine was dissolved in 0.5% methylcellulose 15 (M7140, Sigma-Aldrich, Denmark) in 0.9% NaCl. Siramesine treatment (100 mg/kg) was administered orally using a stomach tube. Control animals received 0.5% methylcellulose 15 in 0.9% NaCl. The treatment included biweekly treatment, initiated 1 week after tumor implantation for U87 implanted mice and 2 weeks after implantation for T78 implanted mice. U87 mice were euthanized after 1 week of treatment, whereas T78 mice were euthanized after 6 weeks of treatment.

The mice were euthanized at the same time point. When symptoms were observed as described below in the first mice, all mice were sacrificed to be able to compare the volumes among groups. The brains were removed immediately after death and fixed in 4% formaldehyde for 48 h. Before paraffin embedding the brains were divided by coronal sections (1 mm). Subsequently, brain sections were cut on a microtome (3 μM), and stained with H&E as well as Vimentin immunohistochemical staining as described previously. The tumor volume was determined using the digital software NanoZoomer Digital Pathologi, NDP viewer (Hamamatsu).

### Ethics

The official Danish ethical review board named the Regional Scientific Ethical Committee of the Region of Southern Demark approved the use of human glioma tissue (permission J. No. S-VF-20040102) in the current study. Written informant consent was obtained from all participants.

The use of animals for organotypic brain slice cultures was approved by The Animal Experiments Inspectorate in Denmark (permission J. No. 2008/561-1572). The rats (newborn wistar rats, Taconic Denmark, *n* = 60; 4–6 slice cultures were obtained per rat) were decapitated and the brains were removed.

The use of animals for glioblastoma mice xenografts were approved by The Animal Experiments Inspectorate in Denmark (permission J. Nr. 2013-15-2934-00973). Mice (Female Balb c nu/nu mice 7–8 weeks, Taconic Denmark, *n* = 42) were anesthetized by a subcutaneous injection with a mixture of hypnorm and dormicum (0.12 ml/10 g). The mice were euthanized in a carbon dioxide chamber upon symptoms such as weight loss (20% loss of body weight) and general poor state including lethargy, hunched posture and failure to groom. The animals were housed according to national guidelines (National declaration for animal experiments 2013), and had free access to food and water.

### Statistics

Data following a Gaussian distribution was analyzed using one-way ANOVA with Dunnett’s post test to compare treated cultures with control cultures. Non-parametric data was analyzed using Kruskal-Wallis with Dunn’s post test to compare the difference in the sum of ranks between two columns. Statistical significance was defined as *P < 0.05, **P < 0.01, ***P < 0.001. EC50 values were estimated by nonlinear regression. The Pearson correlation was calculated to quantify the association between the two variables, WST-1 and LDH. Tumor volume was compared using unpaired *t*-test. All statistics were carried out using Graph Pad Prism 5.0 (Graphpad Software, San Diego California USA).

## Results

### Siramesine-induced cell death in human glioma cell lines

**Fig. 1:**
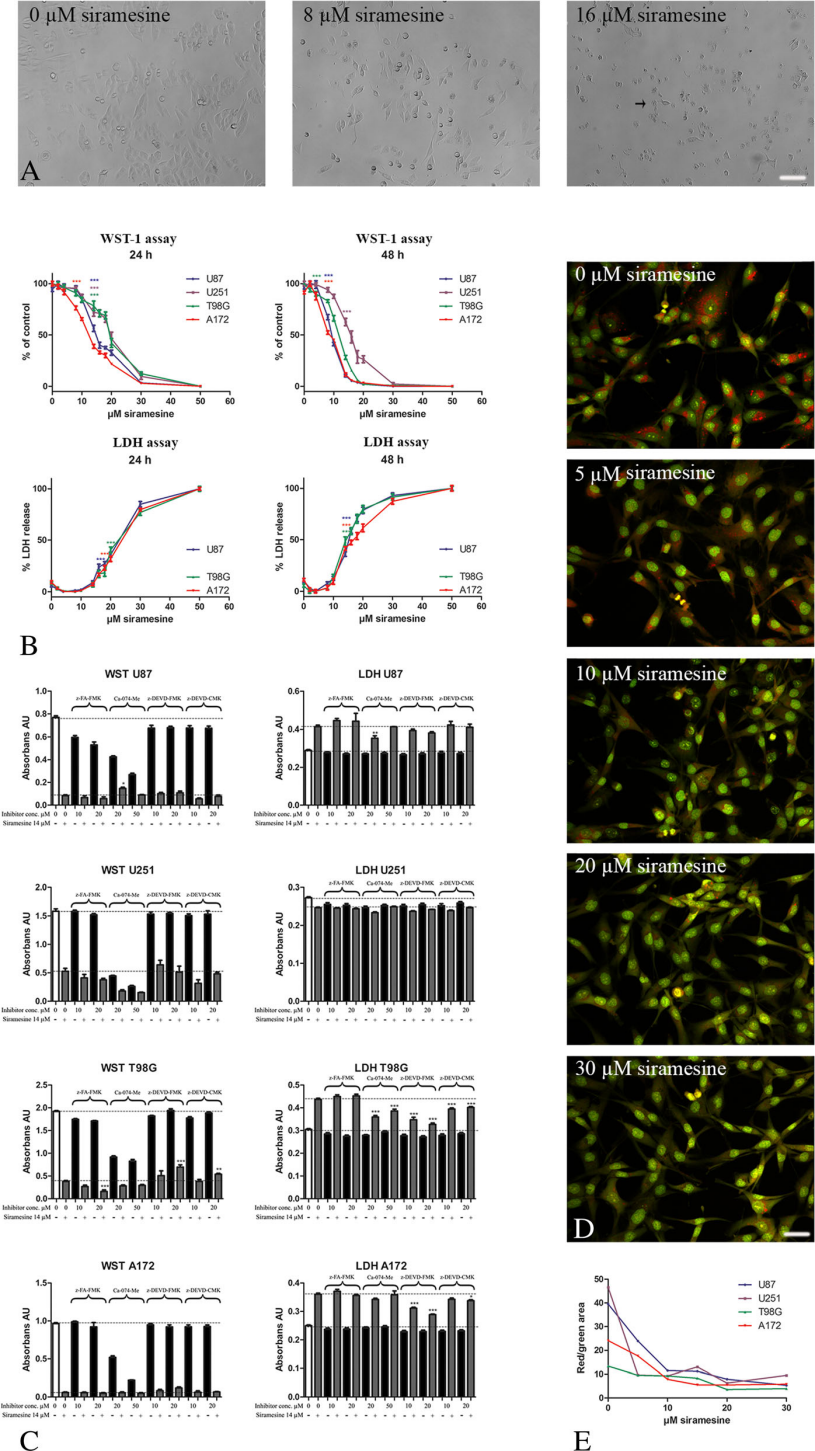
Effect of siramesine in cell lines. The standard human glioma cell lines U87, A172, T98G and U251 were grown as adherent cell cultures and exposed to siramesine. **a** Images of the glioma cell line T98G showed cells which rounded up and shrinked upon siramesine exposure (*arrow*). **b** Upon exposure to siramesine (0–50 μM) cell proliferation (WST-1 assay) cell death (LDH assay) was measured. U87 and A172 were more sensitive towards siramesine compared to U251 and T98G after both 24 and 48 h as seen in both the WST-1 assay (*upper panel*) and the LDH assay (*lower panel*). **c** Inhibitors of cathepsins (z-FA-FMK and Ca-074-Me) and caspases (z-DEVD-FMK and z-DEVD-CMK) were used to identify mediators of siramesine-induced cell death. Cell proliferation (WST-1 assay) and cell death (LDH assay) was measured for all four cell lines, suggesting differential mechanisms in the different cell lines. **d**-**e** Lysosomal involvement was investigated with acridine *orange* which accumulates in acidic cellular compartments, primarily in lysosomes resulting in *red fluorescence*. Acridine *orange* staining in the glioma cell lines appeared as *red* dot-like staining corresponding to the presence of intact lysosomes (0 μM siramesine). Confocal imaging identified loss of red fluorescence in the lysosomes upon siramesine exposure in all of the glioma cell lines after only 1 h of exposure to siramesine (5–30 μM). This suggested that siramesine exposure lead to compromised/ruptured lysosomal membranes. Scalebar 100 μm (**a**), Scalebar 50 μm (**e**). Data are displayed as mean values ± SEM, and *P < 0.05, **P < 0.01, ***P < 0.001 were assessed by one-way ANOVA. AU, arbitrary units

The human glioma cell lines U87, U251, T98G and A172 were exposed to siramesine (0–50 μM). Many cells detached, rounded up and shrinked and some cells appeared with fragmented nuclei resembling apoptotic bodies suggesting apoptosis-like cell death as shown for T98G (Fig. 1a, indicated by arrow). The cell proliferation was significantly reduced after 24 and 48 h when measured with the WST-1 proliferation assay (Fig. 1b, upper panel). The EC50 values (Additional file 1: Table S1) suggested U87 and A172 to be most sensitive towards siramesine. An increase in LDH release was seen in U87, T98G and A172 at slightly higher concentrations than observed in the WST-1 assay (Fig. 1b, lower panel). No LDH release was measured for the cell line U251. The pattern of LDH release was the same after 24 and 48 h, but the effects were more pronounced after 48 h. When correlating the data from the WST-1 assay and the LDH assay, correlation coefficients near −1 for U87, T98G and A172 (Additional file 2: Figure S2) were found, suggesting an almost linear correlation between the two assays. The LDH assay, however, seemed to be less sensitive than the WST-1 assay.

### Cathepsin or caspase dependent cell death

Inhibition of cathepsins and caspases showed different results for the different glioma cell lines used (Fig. 1c). For U87, inhibition of cell proliferation and cell death was obtained by the cathepsin B inhibitor Ca-074-Me in both the WST-1 and LDH assay, whereas no inhibition of the caspase inhibitors were observed. Cathepsin and caspase inhibitors showed no protection against cell death for U251, neither in the WST-1 assay nor in the LDH assay. Proliferation and cell death in T98G was inhibited by both the cathepsin B inhibitor Ca-074-Me and the caspase-3 and-7 and caspase 3 inhibitors, z-DEVD-FMK and z-DEVD-CMK, respectively. In the WST-1 assay the caspase inhibitors inhibited proliferation at high concentrations only. In the LDH assay the cathepsin B inhibitor as well as the caspase inhibitors at both concentrations inhibited cell death. For A172 cell proliferation measured with WST-1 was unaffected whereas cell death in the LDH assay was inhibited by the caspase-3 and −7 inhibitor z-DEVD-FMK and by 20 μM of the caspase 3 inhibitor z-DEVD-CMK. Unexpectedly, when measuring the cell proliferation using the WST-1 assay, the cathepsin inhibitor Ca-074-Me decreased cell proliferation significant without addition of siramesine. Whether this phenomenon was due to an effect of the inhibitor on the cells or interference with the assay is not known. However, when using the LDH assay this was not observed.

### Siramesine induced lysosomal changes in pH

The glioma cell lines, U87, U251, T98G and A172, stained with acridine orange and exposed to siramesine (5–30 μM) showed a decrease in red staining and an increase in green staining suggesting changes in lysosomal pH (Fig. 1d–e).

### Cell death in GSS cultures

**Fig. 2:**
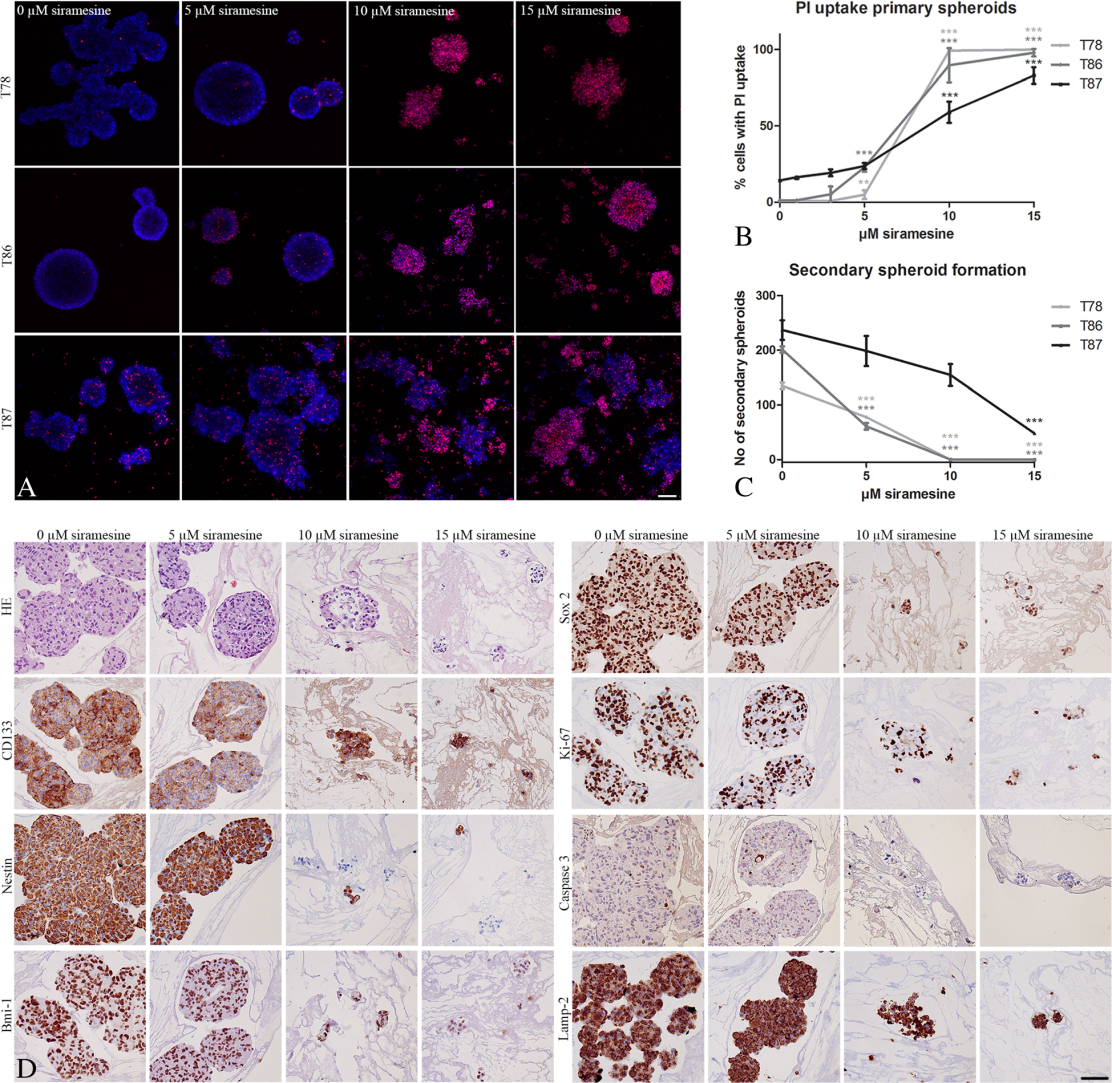
Effect of siramesine on patient-derived spheroid cultures. The glioblastoma stem cell-like containing spheroid (GSS) cultures T78, T86 and T87 were exposed to siramesine (0–15 μM) for 24 h. **a–b** The dye propidium iodide (PI) (*red fluorescence*) enters dead and dying cells and was used to identify cell death in spheroids. PI uptake in all three GSS cultures was already seen by 5 μM siramesine and was pronounced at 10–15 μM siramesine. Hoechst 33324 staining (*blue fluorescence*) was used to stain all cells to be able to calculate a percentage of PI uptake per spheroid. **c** Formation of secondary spheroids from siramesine-exposed primary spheroids was reduced for all three GSS cultures already by 5 μM siramesine. **d** After siramesine exposure of T78 primary spheroids, the spheroids were fixed and paraffin embedded for histology. H&E staining and immunohistochemical staining with CD133, Nestin, Bmi-1, Sox 2, Ki-67, LAMP-2 and caspase 3 of 3 μm histological sections were performed. Only small fragments and single cells were found at concentrations of 10 and 15 μM, suggesting induction of pronounced cell death by siramesine. However, CD133 was expressed in both control spheroids and in siramesine treated spheroids. Nestin was expressed in control spheroids and spheroids exposed to 5 μM siramesine whereas some spheroid residues at 10 and 15 μM had lost the nestin expression. Bmi-1 was expressed in control spheroids and in the siramesine exposed spheroids and the same pattern was seen for Sox2, Ki-67, Caspase 3 and Lamp-2. These staining thus suggested a potential for recurrence. Scalebar 100 μm (**a** and **d**). Data are displayed as mean values ± SEM, and *P < 0.05, **P < 0.01, ***P < 0.001 were assessed by one-way ANOVA

GSS cultures treated with 10 and 15 μM of siramesine showed disintegration of spheroids, and often cellular shrinkage (Additional file 3: Figure S3). Using confocal microscopy a significant PI uptake was observed already at 5–10 μM siramesine (Fig. 2a–b).

### Secondary spheroid formation assay

Formation of secondary spheroids was seen for all GSS cultures after treating primary spheroids with siramesine (Fig. 2c). The number of new spheroids formed for T78 and T86 were already significantly lower at 5 μM siramesine, whereas the number of spheroids for T87 was significantly lower after treatment with 15 μM siramesine.

### Immunohistochemical staining of siramesine exposed GSS cultures

H&E staining of histological sections of GSS cultures revealed shrinkage of cells and disintegration of the spheroids at concentrations of 5–10 μM (Fig. 2d). In general GSS cultures expressed all chosen stem cell markers but different levels of expression were found (images only shown for T78, Fig. 2d). In cultures exposed to siramesine, CD133 expression was preserved for both surviving T78 and T87 cells, whereas it disappeared for T86 at 15 μM siramesine. In spheroids exposed to 10 and 15 μM siramesine a decrease in Nestin expression was found for all three spheroid cultures. For T78 and T87, a decrease in Bmi-1 staining intensity was seen in the spheroids exposed to especially 15 μM. No Bmi-1 expression was seen in T86 spheroids. A small decrease in Sox2 expression was seen for T86 and T87 upon exposure to 15 μM siramesine.

The proliferation marker Ki-67 was expressed in all cell lines and expression was preserved in the cells upon siramesine treatment.

The expression of caspase 3 was found in control cultures in a few cells in T78 spheroids, none in T86 and in a number of cells in T87 spheroids. Upon siramesine exposure only a few cells were found positive in T78, whereas an increase in positive cells was found in T86 spheroids already at 5 μM. The expression in siramesine exposed T87 spheroids resembled the control cultures. Lamp-2 was expressed in both control and siramesine exposed spheroids.

### Spheroid migration assay

**Fig. 3:**
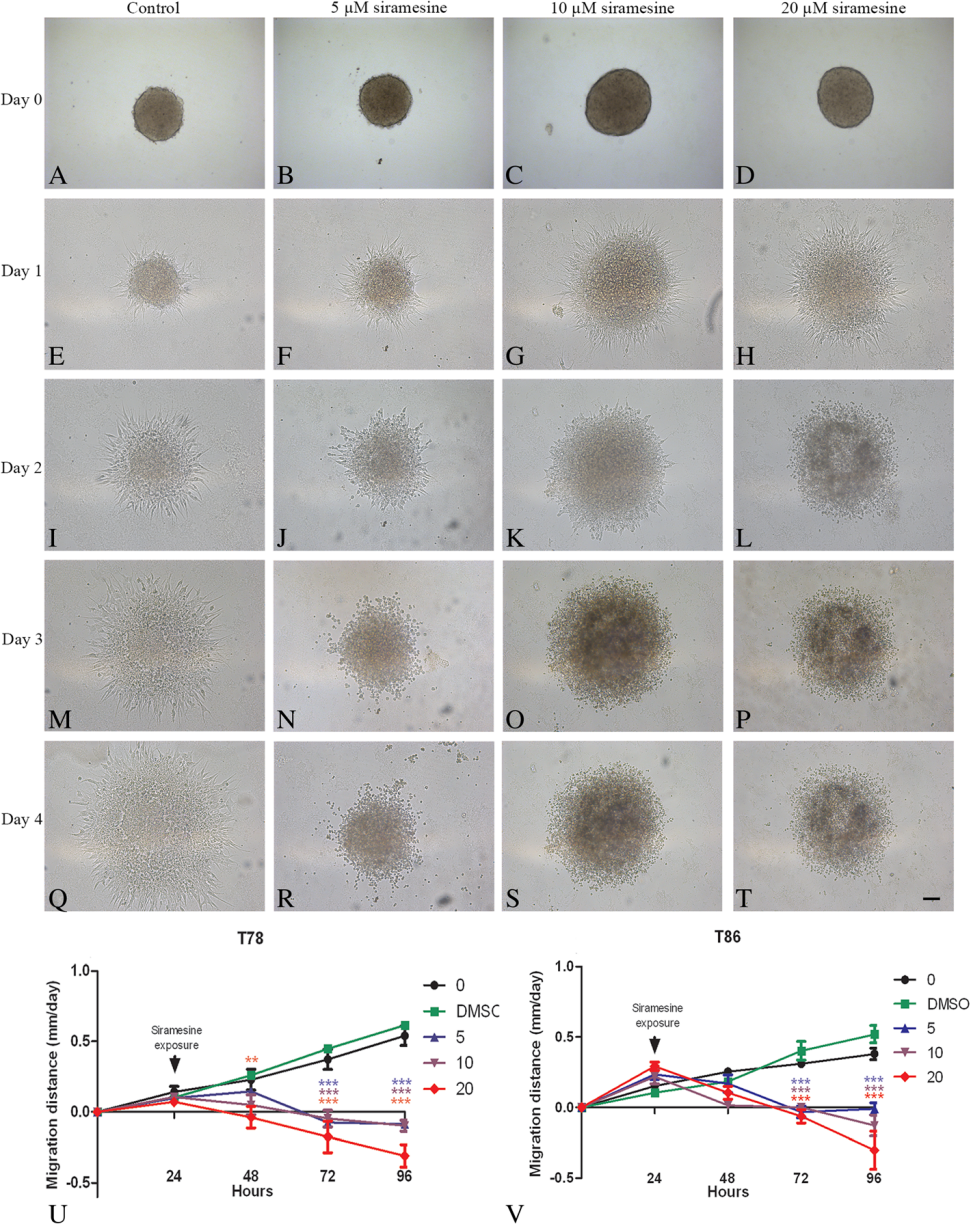
Flat surface spheroid migration assay. The glioblastoma stem cell-like containing spheroid (GSS) culture T78 and T86 (images not shown) were allowed to migrate for 1 day before exposure to siramesine (0–20 μM). **a, e, i, m, q** Control spheroids revealed a pronounced migration. Siramesine reduced migration in T78 spheroids at all concentrations; **b, f, j, n, r** 5 μM, **c, g, k, o, s** 10 μM, and **d, h, l, p, t** 20 μM. **u** The spheroid diameter including all migrating cells was measured at indicated time-points. T78 spheroids exposed to 20 μM siramesine showed a significantly reduced migration distance already 1 day after exposure (in total 48 h after start of experiment), compared to control spheroids. **v** The migration distance was significantly reduced in T86 spheroids 2 days after siramesine exposure (in total 72 h after start of experiment) at all concentrations. Control cells received culture medium or DMSO (images not shown) both without siramesine. Scalebar 100 μm (**a–t**). Data are displayed as mean values ± SEM, and **P < 0.01, ***P < 0.001 were assessed by one-way ANOVA

**Fig. 4:**
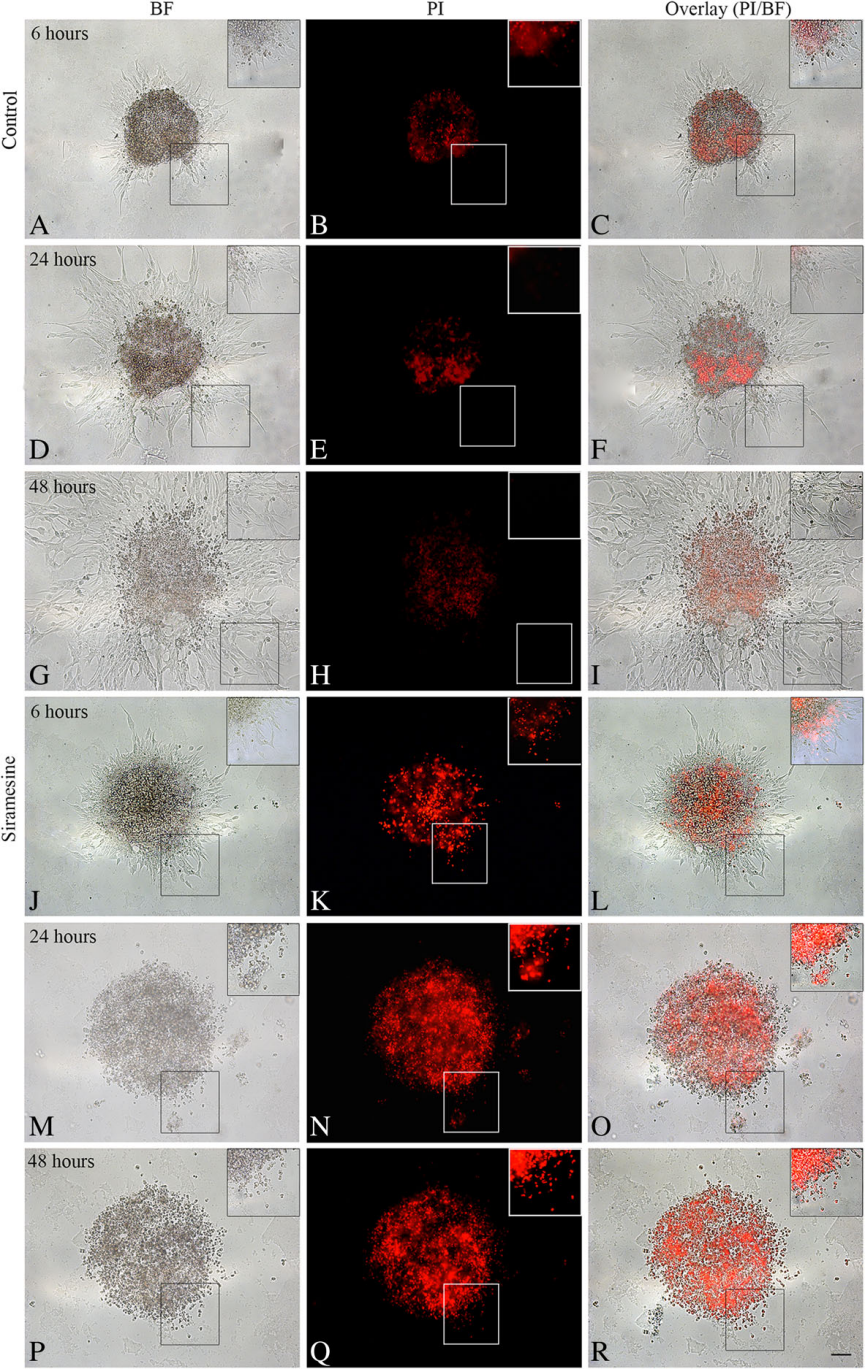
Propidium iodide uptake in flat surface spheroid migration assay. The glioblastoma stem cell-like containing spheroid (GSS) culture T78 were allowed to migrate for 1 day before exposure to 10 μM siramesine, while being incubated with the dye propidium iodide (PI), which enters dead and dying cells. **a–i** In control spheroids, there was a weak central PI uptake but no uptake was observed in the migrating cells. **j–l** In siramesine-exposed spheroids, PI uptake suggested siramesine-induced cell death in the central spheroid itself but also in the migrating cells already 6 h after exposure. **m–r** After 24 and 48 h of exposure a pronounced PI uptake was found both in the spheroid and in the migrating cells. Control cells received culture medium or DMSO (images not shown) both without siramesine. Scalebar 100 μm

T78 spheroid migration was rapidly influenced by siramesine exposure (Fig. 3a–t). Already 1 day after exposure, spheroids receiving 20 μM of siramesine showed a significant decrease in migration distance (*P* < 0.001) (Fig. 3u). In T86 spheroids, a significant decrease in cell migration was found for all exposed groups 2 days after exposure (*P* < 0.001) (Fig. 3v). Cell death in the siramesine exposed cultures was visualized using PI (Fig. 4). Control spheroids showed a diffuse PI uptake but no uptake in the migrating cells was observed throughout the experiment (Fig. 4b–c, e–f and h–i). Siramesine appeared to induce cell death in the migrating cells already 6 h after exposure, but also limited cell death in the central part of the spheroids (Fig. 4k–l). The PI uptake increased after 24 and 48 h after exposure (Fig. 4n–o and q–r).

### PI uptake in brain slice cultures

**Fig. 5:**
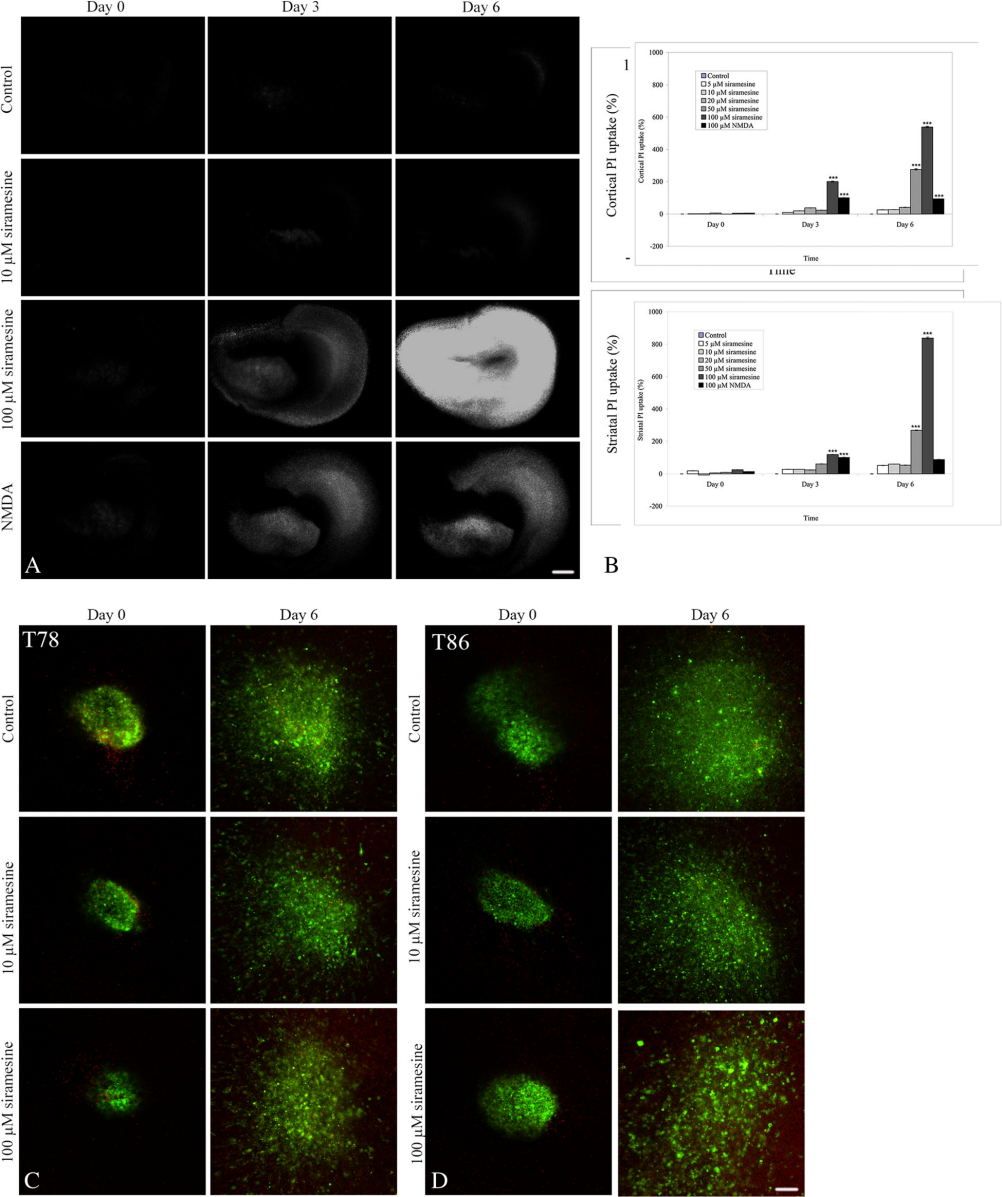
Effects of siramesine in brain slice cultures with spheroids. Spheroids were implanted into organotypic corticostriatal brain slice cultures and exposed to siramesine. **a** Initially, organotypic corticostriatal brain slice cultures without spheroids were exposed to siramesine and the uptake of propidium iodide (PI) was used to visualize potential siramesine-induced death of normal cultured brain tissue. No PI uptake was seen in control cultures and in cultures exposed to 10 μM siramesine. A pronounced PI uptake was observed with 100 μM siramesine, PI uptake at this concentration exceeded cell death in the N-Methyl-D-aspartate (NMDA) treated cultures used as positive control. NMDA is an excitatory amino acid inducing pronounced neuronal cell death in high concentrations. **b** Densitometric measurements of PI uptake in both cortex and striatum revealed significant cell death already at day 3 when the brain slice cultures were exposed to both 50 and 100 μM siramesine. Data was normalized to NMDA-induced cell death at day 3 set to 100%. **c–d** Both T78 (**c**) and T86 (**d**) spheroids were implanted into brain slice cultures. The spheroids were labelled with DiO (*green*) before implantation, whereby tumor cell invasion into the brain slices could be monitored by confocal microscopy. Confocal z-stacks were recorded before (day 0) and 6 days after (day 6) 10 and 100 μM siramesine exposure. Tumor cell invasion was found in controls and at both siramesine concentrations at day 3 and 6 in cultures implanted with both T78 and T86 spheroids. No cell death as visualized by PI uptake was present in spheroids and invasive cells (*yellow* - overlap between DiO (*green*) and PI (*red*)). Results were confirmed by histology in Fig. 6. Control cells received culture medium or DMSO (images not shown) both without siramesine. Scalebar 600 μm (**a**), Scalebar 100 μm (**c–d**). Data are displayed as mean values ± SEM, and **P < 0.01, ***P < 0.001 were assessed by one-way ANOVA

Concentrations of siramesine between 5 and 20 μM did not induce any cell death in the brain tissue (Fig. 5a–b), but at concentrations of 50 and 100 μM, PI uptake was significantly increased in both cortex and striatum (Fig. 5a–b). Concentrations of 100 μM (Fig. 5a–b) induced a high uptake of PI exceeding the uptake in the positive control cultures treated with the glutamate receptor agonist NMDA (Fig. 5a–b) suggesting a pronounced cell death in the brain slice cultures.

### PI uptake in siramesine treated co-cultures

No PI uptake in the T78 (Fig. 5c) and T86 (Fig. 5d) implanted spheroids was observed in the confocal images. At day 6, however, in the cultures exposed to 50 and 100 of μM siramesine, extensive PI uptake was seen in the brain slice cultures (Fig. 5c–d only shown for 100 μM siramesine) but not in the spheroids.

In order to test whether the low PI uptake in implanted spheroids where due to limited diffusion of siramesine through the membrane into brain slice cultures, DiO labelled spheroids were placed directly on the membrane. A significant PI uptake in the spheroids was found confirming the diffusion potential of siramesine across the membrane (Additional file 4: Figure S4).

### Marker expression in siramesine exposed co-cultures

**Fig. 6:**
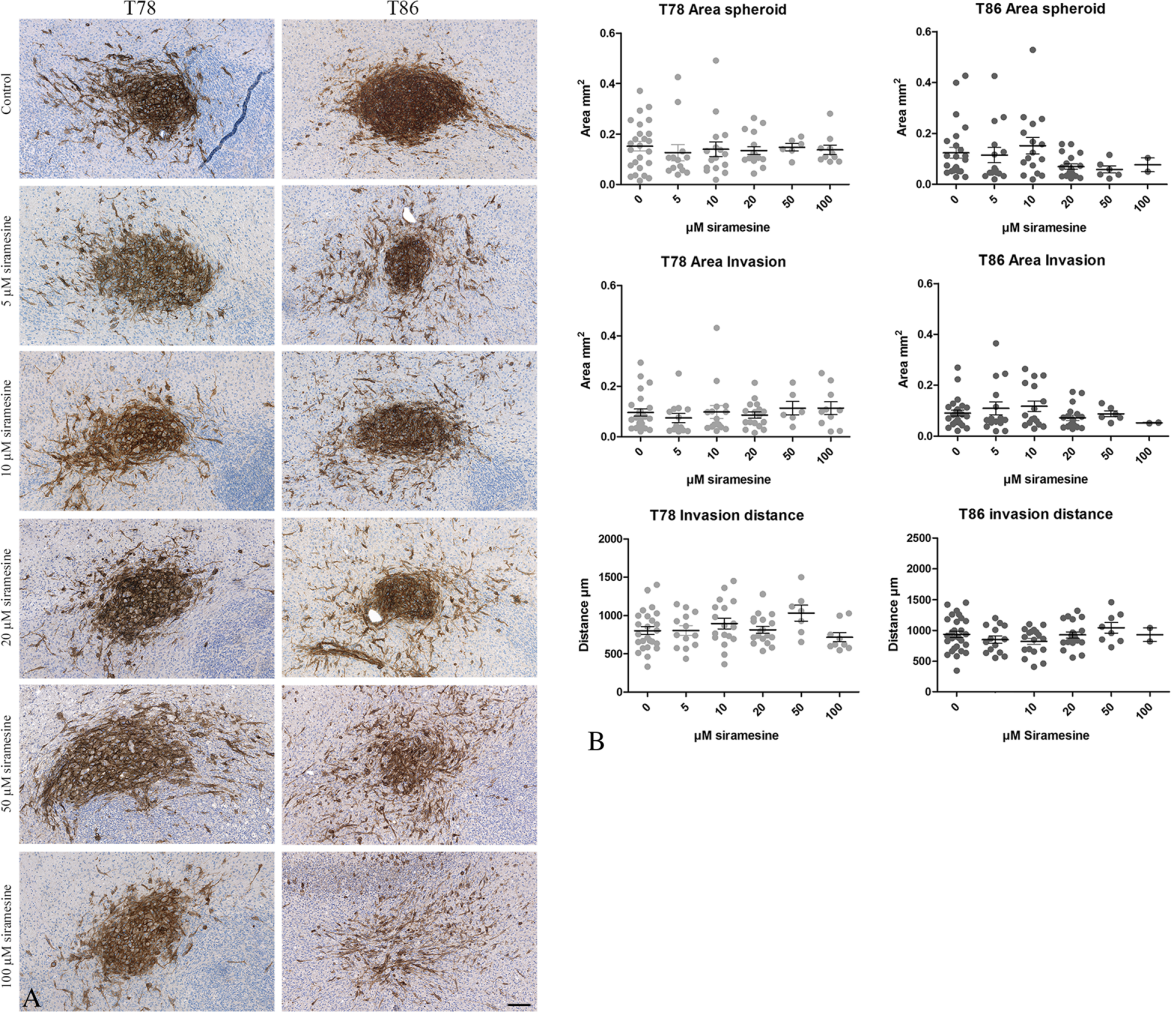
Immunohistochemical detection of tumor cells in the brain slice cultures. After siramesine exposure of organotypic corticostriatal brain slice cultures being implanted beforehand with spheroids, these co-cultures were fixed, paraffin embedded, sectioned (3 μm) and immunohistochemically stained with anti-human specific CD56 in order to identify the tumor cells. For comparison these co-cultures were scanned to monitor invasion and cell death during exposure as illustrated in Fig. 5. **a** Invasion of CD56 positive human tumor cells from T78 and T86 spheroids into the surrounding brain tissue was observed in 3 μm histological sections in all cultures and at all concentrations of siramesine. **b** No difference in spheroid size, invasion area and distance was found between control cultures and cultures being exposed to different concentrations of siramesine. The “invasion area” was defined as the area of cells, which had detached from the spheroids and invaded into the brain tissue. The “invasion distance” was defined as the maximal distance measured to an invasive cell. Control cells received culture medium or DMSO (images not shown) both without siramesine. Scalebar 100 μm (**a**). Data are displayed as mean values ± SEM, and **P < 0.01, ***P < 0.001 were assessed by one-way ANOVA

Immunohistochemical staining with anti-human CD56 was used to identify the spheroids and the invasive cells upon implantation of T78 and T86 into the brain slice cultures (Fig. 6a). No differences in the tumor migration area or distance were found (Fig. 6b), however, a tendency towards a change in morphology from cells being elongated to being more rounded cells were seen in cultures exposed to 100 μM siramesine (Fig. 6a and Additional file 5: Figure S5 shown for T78). When exposing the co-cultures to 100 μM Siramesine, 5 out of 12 cultures implanted with T78 disintegrated upon paraffin embedding and for T86 this number was even higher loosing 10 out of 12 cultures. The surviving cultures were probably less affected by siramesine thus the pictures shown for 100 μM might not reflect the disintegrated cultures. Confocal images of disintegrated cultures showed a major PI uptake in the brain tissue and a small increase in cell death in the spheroids.

### Glioblastoma xenografts

**Fig. 7:**
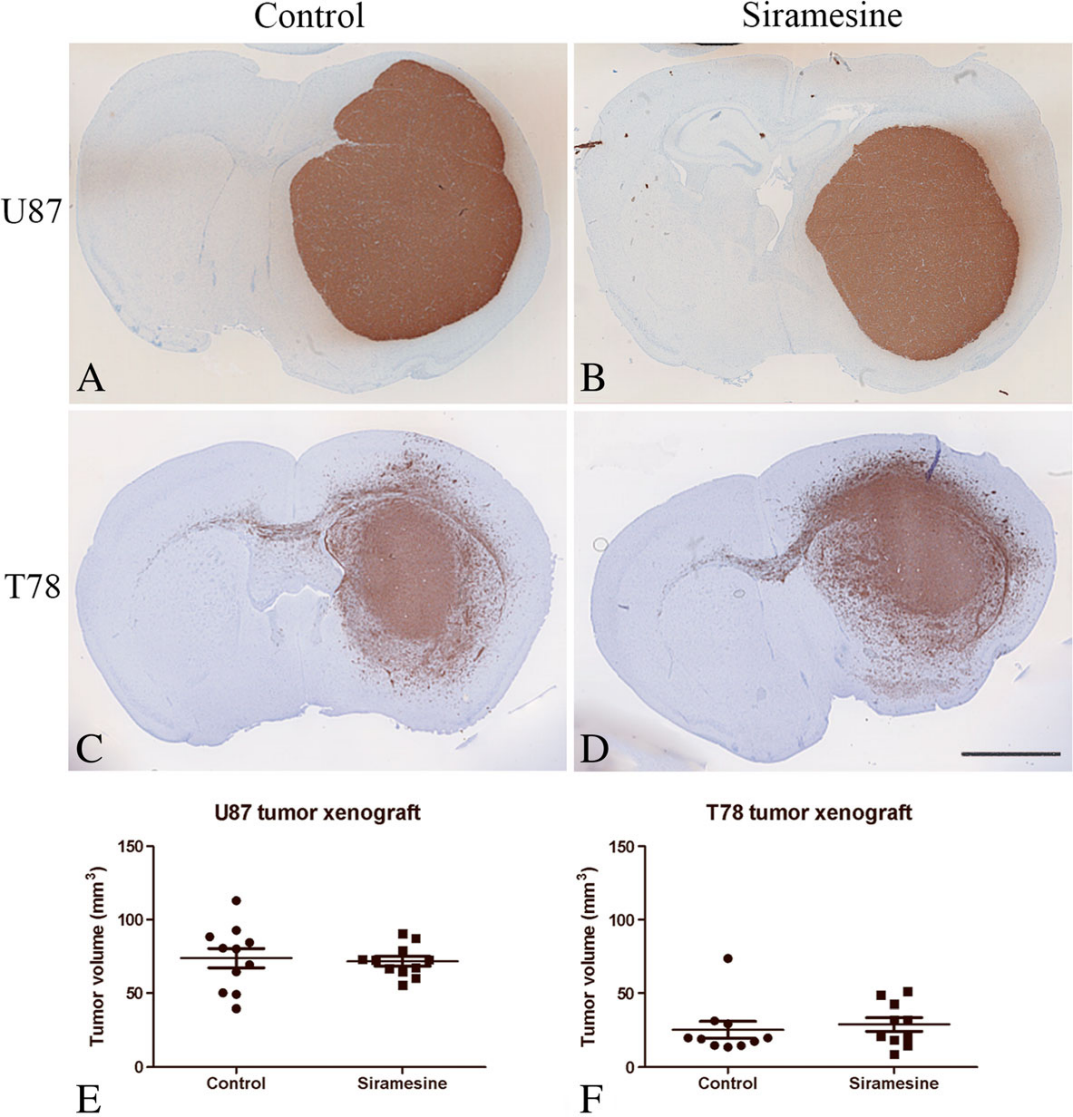
Glioblastoma xenograft. Tumor xenografts were generated by injecting tumor cells into the brains of nude Balb/c mice. The mice received 0.5% methylcellulose in 0.9% NaCl (control) or siramesine. After the experiment, the mice brains were fixed, paraffin embedded, sectioned (3 μm) and immunohistochemically stained with anti-human specific CD56 in order to identify the tumor cells. **a–b, e** The standard U87 cell line formed fast growing circumscribed tumors. Siramesine treatment was initiated 1 week after tumor implantation and sustained for 1 week but did not reduce tumor volume (*P* = 0.78). **c–d, f** Patient-derived T78 spheroids formed slower growing invasive tumors. Siramesine treatment was initiated 2 weeks after tumor implantation and sustained for a total of 6 weeks but did not reduce tumor volume (*P* = 0.62). Scalebar 2 mm (**a–d**). Data are displayed as mean values ± SEM, statistical significance was assessed students *t*-test. AU, arbitraty units

U87 tumors had a well-defined tumor border and a non-invasive growth pattern comprising the majority of one hemisphere (Fig. 7a–b). T78 tumors displayed a diffuse growth pattern, with marked infiltration into the surrounding brain tissue including the contralateral hemisphere (Fig. 7c–d). Biweekly treatment with siramesine at 100 mg/kg did not reduce tumor volume in U87 implanted animals (*P* = 0.78) (Fig. 7b and e) or T78 tumor xenografts (*P* = 0.62) (Fig. 7d and f). The mice showed no detectable side effects during therapy.

## Discussion

Siramesine was found to have a pronounced effect on standard glioma cell lines and on the more immature cells in the GSS cultures. Cell death in the glioma cell lines was proposed to be both cathepsin and caspase-associated, depending on the cell lines used. The surviving cells in GSS cultures preserved expression of the stem cell markers used, and maintained a high proliferation. In the flat surface migration assay siramesine was efficient but in the spheroid-brain slice culture model and xenograft model siramesine was without effect.

### Effect of siramesine on adherent glioma cell lines

The results suggest that glioblastoma cell lines are sensitive towards siramesine. Siramesine appeared efficient on all cell lines using the WST viability assay. Using the LDH assay, higher concentrations were needed to produce a similar effect. The pronounced effect of siramesine on glioblastoma cells is in line with results obtained by tumor cell lines derived from breast, lung, cervix, prostate and connective tissue [9–11]. By addition of cathepsin and caspase inhibitors to the glioma cell lines, siramesine-induced cell death was suggested to be both cathepsin- and caspase-associated depending on the cell lines investigated. Different results have also been found in other cancers regarding the involvement of caspases in siramesine-induced cell death. It has been found to be both caspase-independent [9] and clearly caspase-dependent [17] but also concentration-dependent [18]. These results might suggest that siramesine stimulates different cell death pathways in different cell lines and settings [19]. A more direct lysosomal involvement was investigated with acridine orange which accumulates in acidic cellular compartments, primarily in lysosomes. Only 1 h after siramesine exposure, a decrease in the staining was seen corresponding to loss of acidity, most likely explained by a compromised lysosomal membrane. The same phenomenon has been described breast cancer [10, 17], which suggested that the changes in pH happens before membrane permeabilization, leakage of the cathepsins to the cytosol and eventually cathepsin mediated cell death.

### Effect of siramesine on GSS cultures

Siramesine induced disintegration of spheroids and a correspondingly increased PI uptake. Together with the H&E staining and immunohistochemistry for the stem cell markers, the more immature tumor cells clearly appeared to be affected by siramesine.. Comparing cell death in the adherent cell lines with the GSS cultures, the adherent cell lines had EC50 values after 24 h between 12.6 and 19.3 μM measured by WST-1 assay whereas EC50 for the GSS cultures was between 4.97 and 8.97 μM measured by PI uptake after 24 h. This suggested that the GSS cultures containing more immature stem-like cells might be even more sensitive towards siramesine than the adherent glioma cell lines. The secondary spheroid formation assay revealed a compromised potential of the treated cells to form new spheroids thereby supporting these results. Although the more immature stem-like cells seemed to be affected, some Ki-67 positive cells was found in the remaining spheroids of all three cultures exposed to 10 and 15 μM siramesine, thereby proposing a potential for recurrence.

### Effect of siramesine on migrating tumor cells

The flat surface spheroid migration assay suggested that siramesine killed migrating glioma cells. PI uptake in migrating tumor cells already detached from the spheroids was seen and there was a significant decrease in spheroid migration distance. Part of this decrease may also be explained by tumor cell death in the spheroid itself. In the organotypic 3D migration assay distance of invasion was not affected by siramesine in any of the cultures. However, some of the migrating tumor cells appeared to have lost their membrane protrusions, appearing as small rounded cells suggesting a stop in migration. These cells were primarily seen at concentrations of 100 μM siramesine being however toxic to the brain tissue and thus irrelevant in a clinical setting. It could be speculated if the lack of effect of siramesine on implanted invasive spheroids was due to a phenotypic migration-related shift making the tumor cells siramesine resistant. A phenotypic shift has previously been described as part of the so-called go-or-grow hypothesis, where cells are shifting from a proliferative to an invasive phenotype [20]. When shifting cells to a substrate supporting migration an overexpression of cell survival genes such as bcl-2 family members, and downregulation of mediators of the apoptotic pathways such as the effector caspases was found in migration glioma cells [21] favouring cell survival. In the same study a reduction of genes involved in proliferation was found suggesting the invasive cells to be less proliferative, hence being a difficult target for chemotherapeutic agents. Siramesine, however, is not believed to target preferentially the proliferative cells, by being both caspase-independent and insensitive to bcl-2 [9–11]. Thus the effect of siramesine should not be reduced on this basis.

The lack of effect on implanted invasive spheroids did not appear to be caused by a compromised delivery of siramesine across the membrane. Supratherapeutic concentrations of siramesine caused significant cell death in brain slice cultures grown on the membranes and in spheroids cultured directly on membranes. Whether the brain tissue limits the concentration of siramesine reaching the tumor cells or whether the phenotype of the invasive cells are responsible for lack of effects remains to be determined. Other studies have investigated the effect of drugs on migrating/invasive cells and suggest – in line with the present study – that 3D migration inside brain tissue is a more challenging model when the aim is to target migrating tumor cells [22, 23]. Being exposed to the drug simvastatin, glioma spheroids confronted with fetal rat brain aggregates showed marked migration of tumor cells into fetal brain tissue, whereas growth and migration was prevented in flat surface migration assay [22]. The same phenomenon was described in another study [23] with a polyamine inhibitor. In line with the results from the spheroid-brain slice culture model, the xenograft model with dosages at 100 mg/kg suggested no anti-tumor effect of siramesine using both the conventional U87 cell line as well a patient-derived glioblastoma spheroid culture. We used a drug dosage based on previous in vivo cancer studies with obtained effect, but since our animals showed no sign of side effects higher drug concentrations might be possible [9, 11, 24]. It could be speculated if the blood–brain-barrier (BBB) prevented siramesine to reach the tumor cells. However, it has previously been shown that siramesine crossed the BBB in mice using both per oral and subcutaneous administration [7]. BBB is associated with impaired delivery of drugs [25] but it has been suggested to be heterogeneously disrupted in glioblastomas being however still intact near the growing edge of the tumor [26]. Earlier initiation of treatment might have been more efficient in our study. One previous study showed anti-tumor effect of siramesine in breast xenografts starting treatment before, simultaneously, or after tumor inoculation [9]. These animals received a daily dosage of siramesine. However, another siramesine study showed anti-tumor effect in breast xenografts with a biweekly treatment schedule being similar to our treatment schedule [11]. Previously a combination treatment with siramesine and the chemotherapeutic drug vincristine showed synergistic cytotoxicity but also individual effect of both drugs in mice breast xenografts [11]. Hence future studies testing a set of drugs causing lysosomal membrane permeabilization might identify new promising candidates to proceed with, both as monotherapy and combined therapies.

## Conclusions

In the present study siramesine killed glioblastoma cells and spheroids in vitro and additionally reduced spheroid formation, thereby suggesting a compromising effect on tumor stemness. The pronounced anti-migratory effect of siramesine in a flat surface migration model could not be reproduced in an organotypic spheroid-brain slice culture model, or in glioblastoma mice xenografts. In conclusion the in vitro results suggest a potential of lysosomal destabilizing drugs in killing glioblastoma cells, but siramesine itself appears to be without effect using in vivo-like in vitro models as well as orthotopic in vivo models.

## Additional files

Additional file 1: Table S1. EC50 values obtained using WST-1 and LDH assays. (PDF 26 kb)
Additional file 2: Figure S2. Correlation between WST-1 and LDH data. U87, T98G and A172 were exposed to siramesine cell proliferation (WST-1 assay) and cell death (LDH assay) was measured. A correlation between the WST-1and LDH data was found. The Pearson correlation coefficients, r, were −0.91, −0.97 and −0.79 for U87, T98G and A172, respectively, all being highly significant (*p* < 0.001). No correlation was found for U251 since the LDH level was unaffected by siramesine. (TIF 56 kb)
Additional file 3: Figure S3. Patient-derived spheroids exposed to siramesine. The glioblastoma stem cell-like containing spheroid (GSS) cultures T78, T86 and T87 were exposed to siramesine (0-15 μM) for 24 h. Light microscopy imaging showed that the spheroids started to disintegrate already at 5–10 μM. Scalebar 100 μm. (TIF 2610 kb)
Additional file 4: Figure S4. Propidium iodide uptake in patient-derived spheroids. In order to evaluate the expected diffusion of siramesine through the membranes used for culturing of spheroid-brain slice co-cultures, spheroids alone were placed directly upon these membranes and exposed to medium with 20 μM siramesine. The medium was present below the membranes similar to the procedure when culturing brain slice cultures. (A) Propidium iodide (PI) uptake on day 6 was detected as both red (arrows) and yellow (arrowheads) fluorescence in both T78 and T86 spheroids compared to control cultures. (B) Measuring PI uptake by using a software classifier identifying red and yellow staining per total area, a significant PI uptake was clearly seen, especially on day 6. Scalebar 100 μm. Data are displayed as mean values ± SEM, and ***P* < 0.01, ****P* < 0.001 were assessed by one-way ANOVA. AU, arbitrary units. (TIF 7565 kb)
Additional file 5: Figure S5. Morphological shape of invasive tumor cells upon siramesine exposure. After siramesine exposure of spheroid-brain slice co-cultures, these were fixed, paraffin embedded, sectioned (3 μm) and immunohistochemically stained with anti-human specific CD56 in order to identify the tumor cells. The images show high magnification of 3 μm thick CD56 stained sections of T78 spheroids implanted into brain slice cultures. (A-B) Control co-cultures were found to have elongated invasive tumor cells. (C-D) Co-cultures exposed to 100 μM siramesine appeared to be have more rounded invasive cells with loss of cell protrusions suggesting a moderate effect of siramesine on invasive tumor cells. Scalebar 100 μm. (TIF 5073 kb)

## Abbreviations

BBB: Blood–brain-barrier
DMSO: Dimethylsulfoxid
GSS: Glioblastoma stem cell-like containing spheroid
H&E: Haematoxylin and Eosin
LDH: Lactate dehydrogenase
PI: Propidium iodide

## References

[1] Stupp R, Mason WP, van den Bent MJ, Weller M, Fisher B, Taphoorn MJ, Belanger K, Brandes AA, Marosi C, Bogdahn U (2005). Radiotherapy plus concomitant and adjuvant temozolomide for glioblastoma. N Engl J Med.

[2] Chen J, Li Y, Yu TS, McKay RM, Burns DK, Kernie SG, Parada LF (2012). A restricted cell population propagates glioblastoma growth after chemotherapy. Nature.

[3] Demuth T, Berens ME (2004). Molecular mechanisms of glioma cell migration and invasion. J Neuro-Oncol.

[4] Boya P, Kroemer G (2008). Lysosomal membrane permeabilization in cell death. Oncogene.

[5] Cirman T, Oresic K, Mazovec GD, Turk V, Reed JC, Myers RM, Salvesen GS, Turk B (2004). Selective disruption of lysosomes in HeLa cells triggers apoptosis mediated by cleavage of Bid by multiple papain-like lysosomal cathepsins. J Biol Chem.

[6] Jaattela M (2004). Multiple cell death pathways as regulators of tumour initiation and progression. Oncogene.

[7] Sanchez C, Arnt J, Costall B, Kelly ME, Meier E, Naylor RJ, Perregaard J (1997). The selective sigma2-ligand Lu 28–179 has potent anxiolytic-like effects in rodents. J Pharmacol Exp Ther.

[8] Heading C (2001). Siramesine H Lundbeck. Curr Opin Investig Drugs.

[9] Ostenfeld MS, Fehrenbacher N, Hoyer-Hansen M, Thomsen C, Farkas T, Jaattela M (2005). Effective tumor cell death by sigma-2 receptor ligand siramesine involves lysosomal leakage and oxidative stress. Cancer Res.

[10] Ostenfeld MS, Hoyer-Hansen M, Bastholm L, Fehrenbacher N, Olsen OD, Groth-Pedersen L, Puustinen P, Kirkegaard-Sorensen T, Nylandsted J, Farkas T (2008). Anti-cancer agent siramesine is a lysosomotropic detergent that induces cytoprotective autophagosome accumulation. Autophagy.

[11] Groth-Pedersen L, Ostenfeld MS, Hoyer-Hansen M, Nylandsted J, Jaattela M (2007). Vincristine induces dramatic lysosomal changes and sensitizes cancer cells to lysosome-destabilizing siramesine. Cancer Res.

[12] Jensen SS, Aaberg-Jessen C, Andersen C, Schroder HD, Kristensen BW (2013). Glioma spheroids obtained via ultrasonic aspiration are viable and express stem cell markers: a new tissue resource for glioma research. Neurosurgery.

[13] Aaberg-Jessen C, Norregaard A, Christensen K, Pedersen CB, Andersen C, Kristensen BW (2013). Invasion of primary glioma- and cell line-derived spheroids implanted into corticostriatal slice cultures. Int J Clin Exp Pathol.

[14] Norregaard A, Jensen SS, Kolenda J, Aaberg-Jessen C, Christensen KG, Jensen PH, Schroder HD, Kristensen BW (2012). Effects of chemotherapeutics on organotypic corticostriatal slice cultures identified by a panel of fluorescent and immunohistochemical markers. Neurotox Res.

[15] Halle B, Marcusson EG, Aaberg-Jessen C, Jensen SS, Meyer M, Schulz MK, Andersen C, Kristensen BW. Convection-enhanced delivery of an anti-miR is well-tolerated, preserves anti-miR stability and causes efficient target de-repression: a proof of concept. J Neurooncol. 2016;126(1):47–55. doi:10.1007/s11060-015-1947-2. 10.1007/s11060-015-1947-2 26428358

[16] Halle B, Thisgaard H, Hvidsten S, Dam JH, Aaberg-Jessen C, Thykjaer AS, Hoilund-Carlsen PF, Schulz MK, Andersen C, Kristensen BW (2015). Estimation of tumor volumes by 11C-MeAIB and 18 F-FDG PET in an orthotopic glioblastoma rat model. J Nucl Med.

[17] Jonhede S, Petersen A, Zetterberg M, Karlsson JO (2010). Acute effects of the sigma-2 receptor agonist siramesine on lysosomal and extra-lysosomal proteolytic systems in lens epithelial cells. Mol Vis.

[18] Zeng C, Rothfuss J, Zhang J, Chu W, Vangveravong S, Tu Z, Pan F, Chang KC, Hotchkiss R, Mach RH (2012). Sigma-2 ligands induce tumour cell death by multiple signalling pathways. Br J Cancer.

[19] Fehrenbacher N, Jaattela M (2005). Lysosomes as targets for cancer therapy. Cancer Res.

[20] Giese A, Loo MA, Tran N, Haskett D, Coons SW, Berens ME (1996). Dichotomy of astrocytoma migration and proliferation. Int J Cancer.

[21] Mariani L, Beaudry C, McDonough WS, Hoelzinger DB, Demuth T, Ross KR, Berens T, Coons SW, Watts G, Trent JM (2001). Glioma cell motility is associated with reduced transcription of proapoptotic and proliferation genes: a cDNA microarray analysis. J Neuro-Oncol.

[22] Gliemroth J, Zulewski H, Arnold H, Terzis AJ (2003). Migration, proliferation, and invasion of human glioma cells following treatment with simvastatin. Neurosurg Rev.

[23] Terzis AJ, Pedersen PH, Feuerstein BG, Arnold H, Bjerkvig R, Deen DF (1998). Effects of DFMO on glioma cell proliferation, migration and invasion in vitro. J Neuro-Oncol.

[24] Petersen NH, Olsen OD, Groth-Pedersen L, Ellegaard AM, Bilgin M, Redmer S, Ostenfeld MS, Ulanet D, Dovmark TH, Lonborg A (2013). Transformation-associated changes in sphingolipid metabolism sensitize cells to lysosomal cell death induced by inhibitors of acid sphingomyelinase. Cancer Cell.

[25] Pardridge WM (2005). The blood–brain barrier: bottleneck in brain drug development. NeuroRx.

[26] Agarwal S, Manchanda P, Vogelbaum MA, Ohlfest JR, Elmquist WF (2013). Function of the blood–brain barrier and restriction of drug delivery to invasive glioma cells: findings in an orthotopic rat xenograft model of glioma. Drug Metab Dispos.

