# Supplementary material for: Effects of the lysosomal destabilizing drug siramesine on glioblastoma in vitro and in vivo
Source: BMC Cancer. 2017 Mar 7;17:178. doi: 10.1186/s12885-017-3162-3 (PMC5341392; doi:10.1186/s12885-017-3162-3)
Supplement: Additional file 1: Table S1. — EC50 values obtained using WST-1 and LDH assays. (PDF 26 kb) [file 12885_2017_3162_MOESM1_ESM.pdf]

S1 Table EC50 values obtained using WST-1 and LDH assays

**Cell line/Time**	**24 hours** <br>**WST-1 assay/LDH assay** <br>**(**µM)	**48 hours** <br>**WST-1 assay/LDH assay** <br>**(**µM)
U87	15.3/23.3	9.2/14.9
U251	19.2/-	15.6/-
T98G	19.3/24.3	11.3/14.2
A172	12.6/24.7	8.7/14.9
