# Supplementary figures and images for: Effects of the lysosomal destabilizing drug siramesine on glioblastoma in vitro and in vivo
Source: BMC Cancer. 2017 Mar 7;17:178. doi: 10.1186/s12885-017-3162-3 (PMC5341392; doi:10.1186/s12885-017-3162-3)

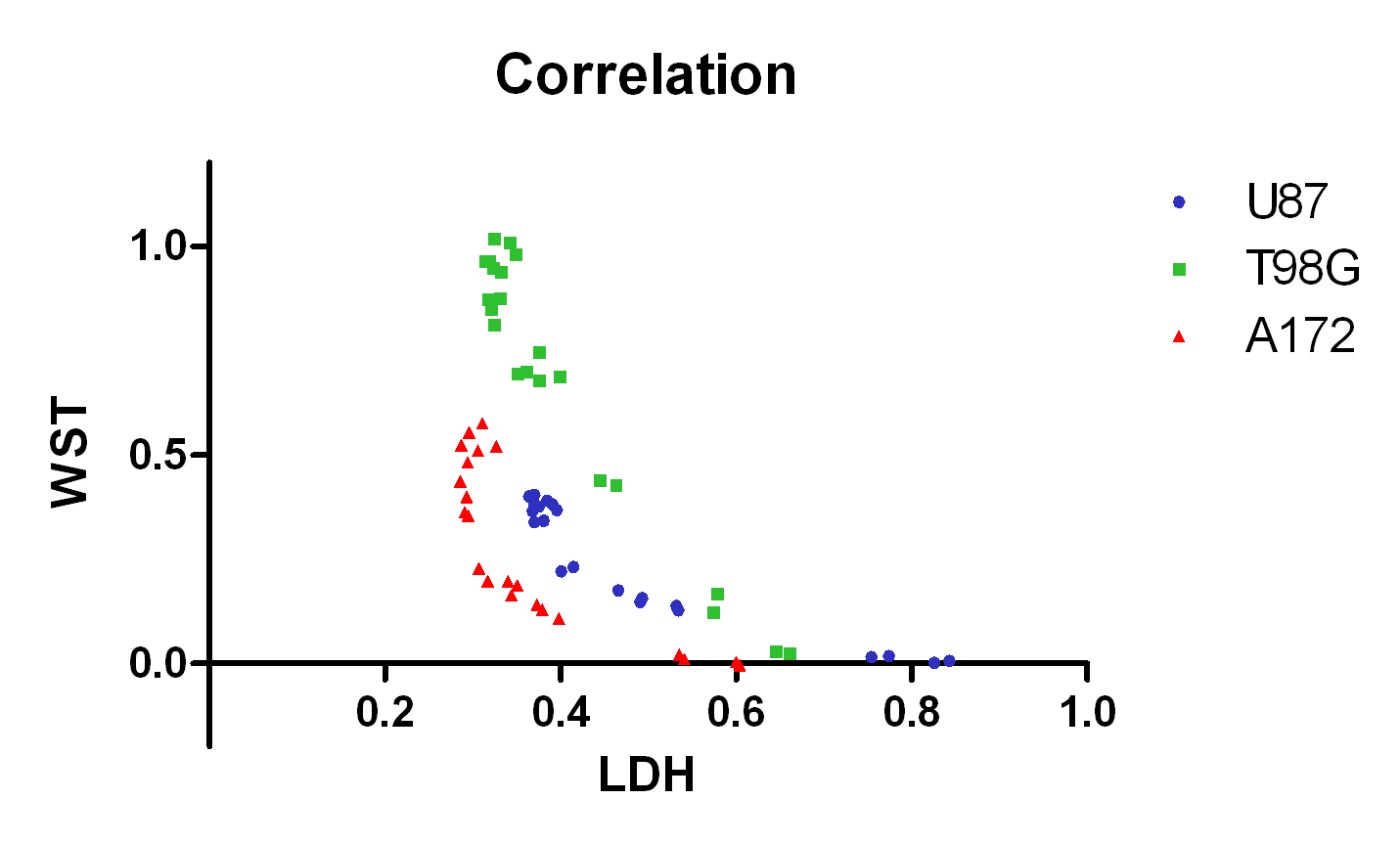

Supplement: Additional file 2: Figure S2. — Correlation between WST-1 and LDH data. U87, T98G and A172 were exposed to siramesine cell proliferation (WST-1 assay) and cell death (LDH assay) was measured. A correlation between the WST-1and LDH data was found. The Pearson correlation coefficients, r, were −0.91, −0.97 and −0.79 for U87, T98G and A172, respectively, all being highly significant (p < 0.001). No correlation was found for U251 since the LDH level was unaffected by siramesine. (TIF 56 kb) [file 12885_2017_3162_MOESM2_ESM.tif]

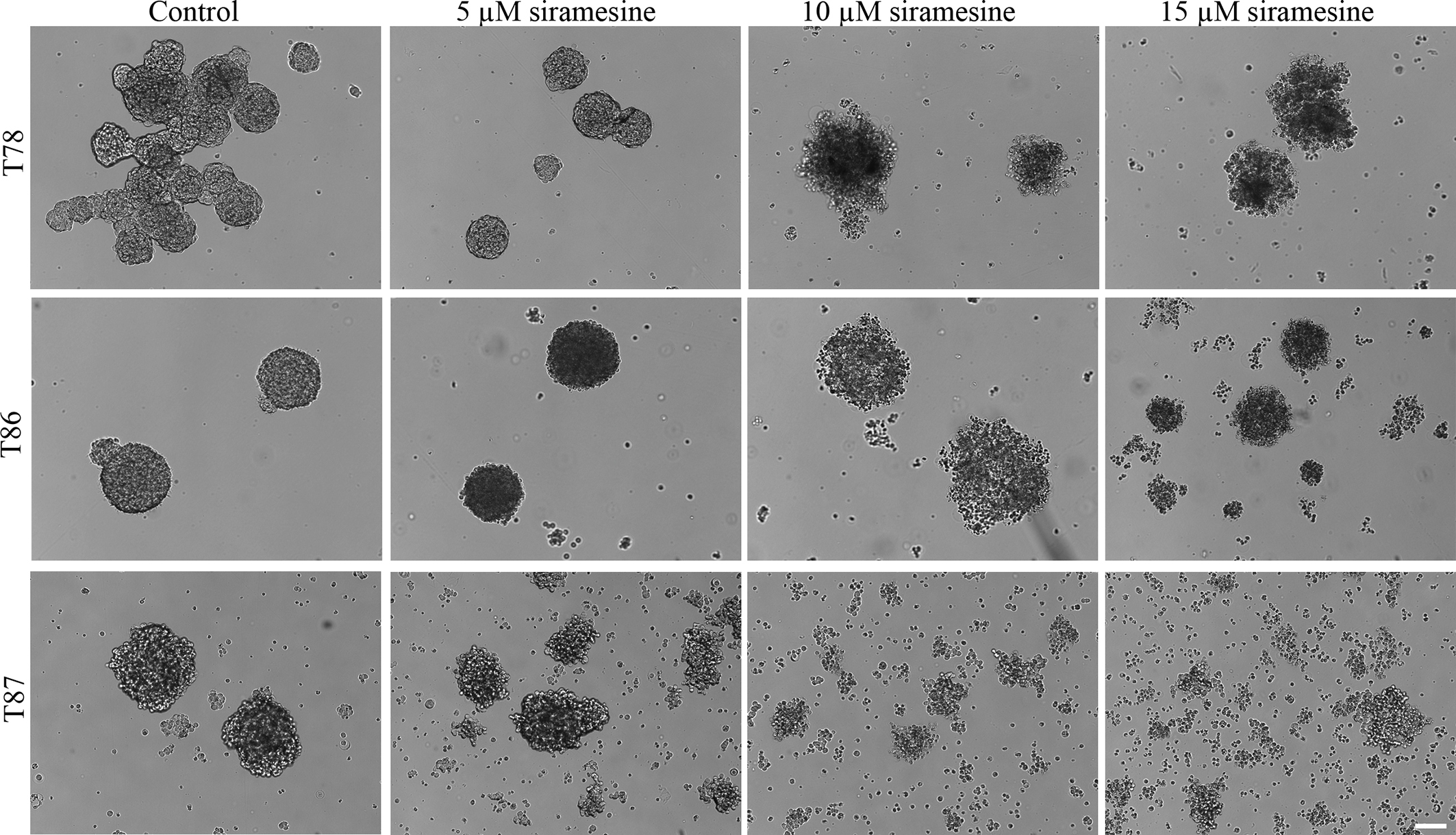

Supplement: Additional file 3: Figure S3. — Patient-derived spheroids exposed to siramesine. The glioblastoma stem cell-like containing spheroid (GSS) cultures T78, T86 and T87 were exposed to siramesine (0-15 μM) for 24 h. Light microscopy imaging showed that the spheroids started to disintegrate already at 5–10 μM. Scalebar 100 μm. (TIF 2610 kb) [file 12885_2017_3162_MOESM3_ESM.tif]

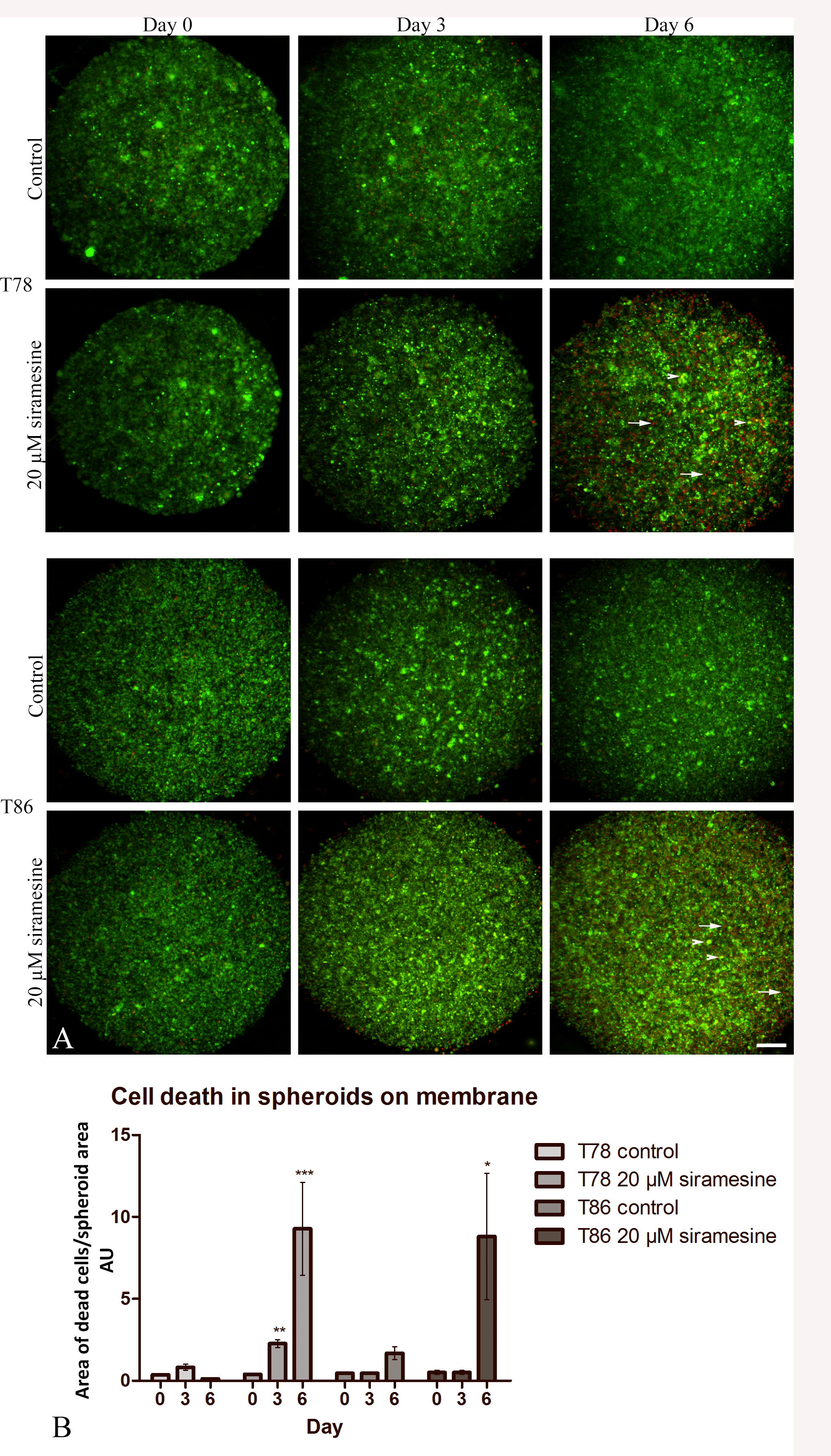

Supplement: Additional file 4: Figure S4. — Propidium iodide uptake in patient-derived spheroids. In order to evaluate the expected diffusion of siramesine through the membranes used for culturing of spheroid-brain slice co-cultures, spheroids alone were placed directly upon these membranes and exposed to medium with 20 μM siramesine. The medium was present below the membranes similar to the procedure when culturing brain slice cultures. (A) Propidium iodide (PI) uptake on day 6 was detected as both red (arrows) and yellow (arrowheads) fluorescence in both T78 and T86 spheroids compared to control cultures. (B) Measuring PI uptake by using a software classifier identifying red and yellow staining per total area, a significant PI uptake was clearly seen, especially on day 6. Scalebar 100 μm. Data are displayed as mean values ± SEM, and **P < 0.01, ***P < 0.001 were assessed by one-way ANOVA. AU, arbitrary units. (TIF 7565 kb) [file 12885_2017_3162_MOESM4_ESM.tif]

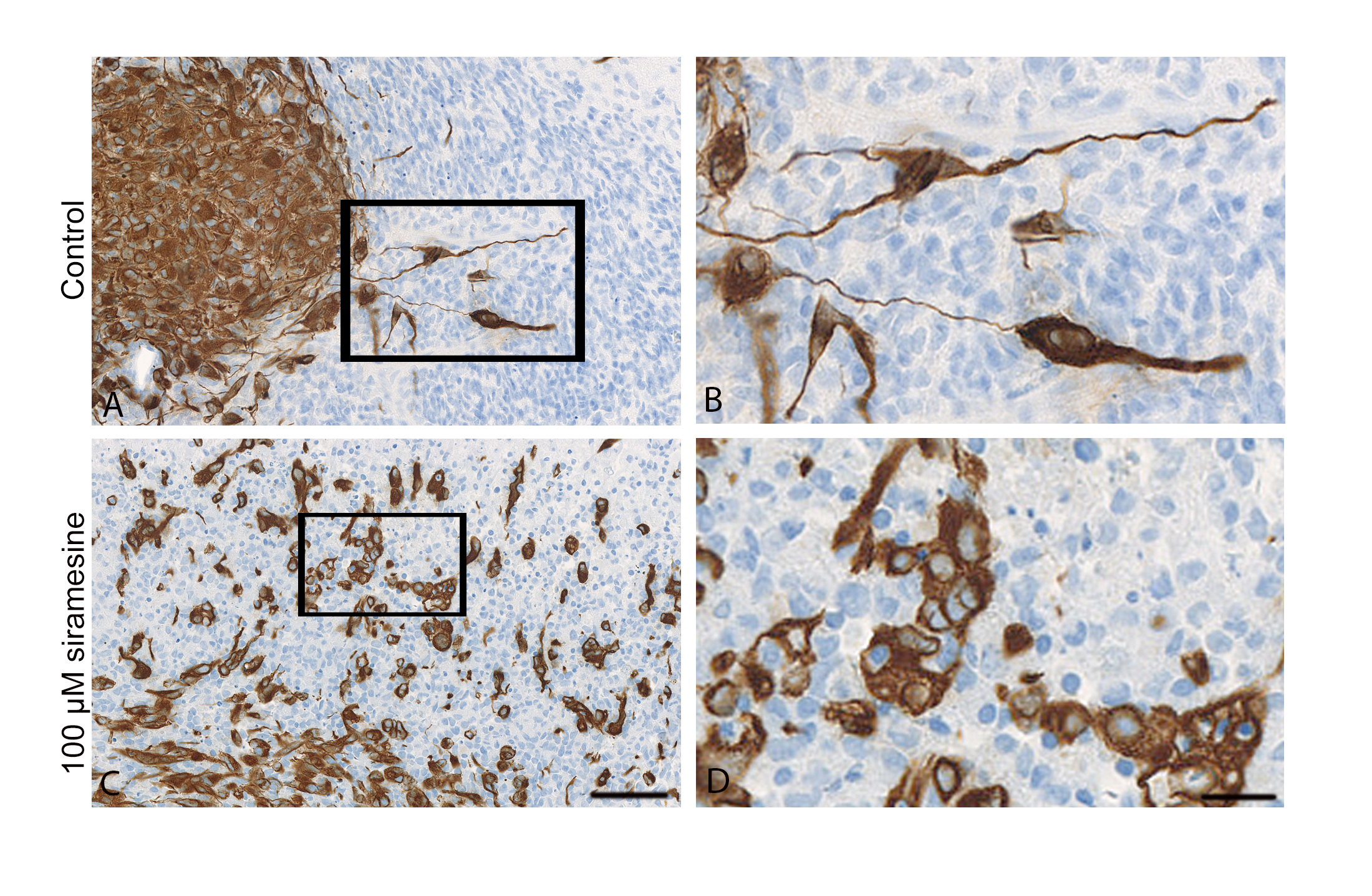

Supplement: Additional file 5: Figure S5. — Morphological shape of invasive tumor cells upon siramesine exposure. After siramesine exposure of spheroid-brain slice co-cultures, these were fixed, paraffin embedded, sectioned (3 μm) and immunohistochemically stained with anti-human specific CD56 in order to identify the tumor cells. The images show high magnification of 3 μm thick CD56 stained sections of T78 spheroids implanted into brain slice cultures. (A-B) Control co-cultures were found to have elongated invasive tumor cells. (C-D) Co-cultures exposed to 100 μM siramesine appeared to be have more rounded invasive cells with loss of cell protrusions suggesting a moderate effect of siramesine on invasive tumor cells. Scalebar 100 μm. (TIF 5073 kb) [file 12885_2017_3162_MOESM5_ESM.tif]
